# Limited access to clean water and sanitation in Mopeia, Mozambique: a description in the context of a cholera outbreak

**DOI:** 10.7189/jogh.15.04197

**Published:** 2025-07-11

**Authors:** Ekhiñe Oroz Torrea, Saimado Imputiua, Nika Gorski, Eldo Elobolobo, Joanna Furnival-Adams, Edgar Jamisse, Patricia Nicolas, Julia Montaña, Vegovito Vegove, Humberto Munguambe, Paula Ruiz-Castillo, Hansel Mundaca, Matthew Rudd, Regina Rabinovich, Francisco Saute, Charfudin Sacoor, Carlos Chaccour

**Affiliations:** 1 Faculty of Medicine, Universidad de Navarra, Pamplona, Spain; 2 Our Lady of Guadalupe Catholic Clinic, Mile 91, Sierra Leone; 3 Centro de Investigaçao em Saúde de Manhiça, Manhica, Mozambique; 4 ISGlobal, Hospital Clínic – Universitat de Barcelona, Barcelona, Spain; 5 Data Brew LLC, Gainesville, Florida, USA; 6 Silver Lining, Manica, Mozambique; 7 Facultat de Medicina i Ciències de la Salut, Universitat de Barcelona, Barcelona, Spain; 8 Harvard T.H. Chan School of Public Health, Boston, Massachusetts, USA; 9 Sewanee: The University of the South, Sewanee, Georgia, USA; 10 CIBERINFEC, Madrid, Spain; 11 Navarra Centre for International Development, Universidad de Navarra, Pamplona, Spain

## Abstract

**Background:**

Inadequate access to safe water, sanitation, and hygiene (WASH) accounts for a high burden of morbidity and mortality in impoverished regions. This is significantly due to infectious diseases and the direct impact on social and economic well-being. The high burden of communicable diseases and malnutrition in Mozambique, as well as high vulnerability to climate change, results in increased risk of WASH-related diseases. Our objective was to describe access to safe water and sanitation practices in Mopeia, a remote rural district in Mozambique.

**Methods:**

The source of data for this analysis is a cross-sectional, demographic survey carried out in Mopeia in 2021 under the Broad One Health Endectocide-based Malaria Intervention in Africa project, a cluster-randomised trial to assess the impact of ivermectin on malaria transmission. The survey was conducted in all households of a sub-population created for the trial, and it included questions about WASH-related practices at the household level.

**Results:**

The results showed that  4200 (56.29%) households have an improved water source at walking distance, which is drastically different to sanitation practices, where 6608 (88.56%) households do not have access to at least one basic sanitation service. Data on water access for Mopeia was similar to that reported in rural contexts in sub-Saharan Africa, yet the district remains off-track from achieving universal safe water coverage in the next few years. Regarding sanitation, the use of unsafe sanitation services is more widespread than in the average rural sub-Saharan Africa (75.00%), with twice as many households (n = 3897, 56.08%) practising open land defecation.

**Conclusions:**

Mopeia is still far from achieving universal safe water and sanitation coverage by 2030, especially in sanitation, and remains prone to outbreaks and has a high burden of WASH-related diseases.

Inadequate access to safe water, sanitation, and hygiene (WASH) services leaves millions vulnerable to many preventable diseases [1]. Contamination of water sources with human and animal faecal matter, limited access to handwashing facilities, and poor environmental management can lead to increased exposure to water- and food-borne diseases, increased human-human or human-animal disease transmission, and a higher number of breeding sites for disease vectors. According to the joint United Nations International Children’s Emergency Fund (UNICEF) and World Health Organization (WHO) 2022 report, one in four people worldwide lacked access to safe drinking water, and nearly half the world’s population still lacked appropriate sanitation services in 2020 [2]. In 2019, the use of unsafe WASH services was associated with the loss of at least 524 000 lives and 37 million disability-adjusted life years in sub-Saharan Africa (SSA), representing 36.0% and 49.5% of global WASH-attributable deaths and disability-adjusted life years lost, respectively [3].

Diarrhoeal diseases account for most WASH-related diseases and are the second most common cause of death in children aged <5 years. According to WHO, there were over one million deaths associated with diarrhoeal diseases in 2019, of which 38% were in SSA [3]. Most moderate-to-severe diarrhoea cases in low-income countries are caused by just two pathogens – rotavirus and *Escherichia coli* – which are both related to poor hygienic conditions. Cholera outbreaks also occur regularly in a few SSA countries and can be associated with high case fatality rates. Neglected tropical diseases, acute respiratory diseases, enteric infections, malnutrition, nosocomial infections and antimicrobial resistance are also associated with inappropriate WASH practices. Not only do these diseases cause significant morbidity and mortality, but they also directly affect economic productivity and perpetuate poverty traps. Better access to WASH facilities reduces health care costs and increases productivity in the workplace. This translates into an estimated USD 5 return on every dollar invested in water and sanitation facilities [1].

One of the Sustainable Development Goals is to achieve access to adequate and equitable sanitation and hygiene for all, and to end open defecation by 2030. Whilst global coverage of appropriate WASH facilities has increased from 47% in 2015 to 54% in 2020, significant variation in progress exists across and within different countries. Efforts to improve the coverage of safely managed drinking water and eliminate open land defecation in SSA need acceleration if we meet the 2030 goals. With the current trends, safely managed drinking water will only reach 37% of the population, and open defecation will not be eliminated in SSA by 2030 [2].

Mozambique is one of the countries with the lowest annual gross domestic product per capita in the world [4]. According to the World Bank, over 60% of the population lives on less than USD 2.15 a day [5]. Some specific characteristics of Mozambique contribute to perpetuate or worsen the burden of WASH-related diseases, such as high rates of HIV, malnutrition and vulnerability to climate-related disasters. In 2022, Mozambique had a similar coverage of at least basic drinking water services compared to SSA in rural (50%) and urban (85%) areas. Around 40% of the Mozambican population had at least a basic sanitation service, and 20% were practising open defecation, which is aligned with the SSA’s average [6]. The basic sanitation coverage varies widely by sub-national region within Mozambique, where few regions approach universal coverage while others lag severely behind [2]. Improving access to safe WASH practices is a crucial intervention to strengthen the health system and improve the national economy [1].

This study took place in Mopeia, a rural district in the Zambezia Province of Mozambique, which has a high burden of WASH-related diseases, including diarrhoea, enteric fevers, and neglected tropical diseases. Our main objective was to describe access to safe water and sanitation practices in Mopeia.

## METHODS

### Study population

**Figure 1:**
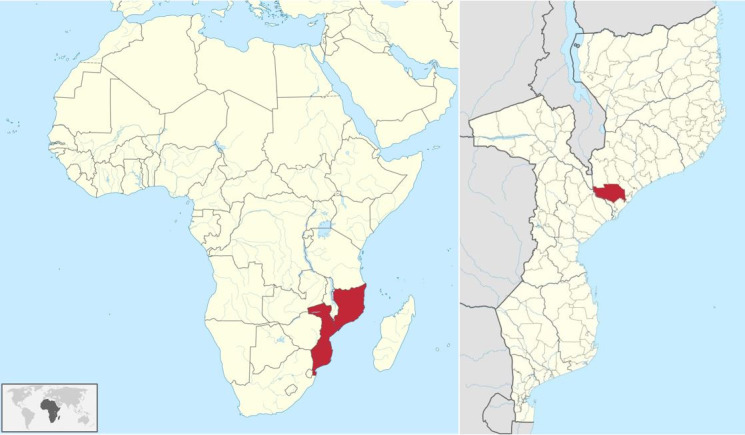
Maps of Mozambique and Mopeia.

Mopeia is one of the 22 districts that make up Zambezia Province in Mozambique (**Figure 1**). It has a surface of 7671 km^2^, with 131 138 inhabitants [7]. From an economic perspective, Mopeia is among the poorest districts in the country, with over 70% of the population subsisting on less than USD 2.15 per day [8]. Mopeia is highly vulnerable to climate change and has experienced frequent flooding, displaced populations, and recurrent road breakdowns in the last few years [9], all of which favour the occurrence of waterborne diseases.

### Data source

The data source of the analysis is a demographic survey carried out in Mopeia in 2021 under the Broad One Health Endectocide-based Malaria Intervention in Africa (BOHEMIA) project [7]. The objective of BOHEMIA was to evaluate the impact of mass drug administration of ivermectin to humans and/or livestock in malaria transmission [7]. Data on the cholera outbreak were extracted from the ad hoc database kept by the district’s health directorate in 2022.

### Study design

In preparation for a clinical trial, we carried out a district-wide mapping and enumeration of all households. A total of 25 550 households and 131 818 people were registered in Mopeia. From this universe, we created a set of 159 study clusters for the trial. We conducted a thorough cross-sectional demographic survey in all the households of this sub-population.

### Demographic survey questionnaire

The survey consisted of a household-level survey answered by the heads of households or their substitutes, and an individual questionnaire for each household member. The answers were not verified by observation at home. Within the census, we collected relevant WASH aspects: demography (number of household members, age, gender, socio-economic level, education, location, distance to health facility), water access (safety of the source, primary source, distance) and sanitation (safety of the sanitation service, place of defecation, surrounding water bodies, information about latrines). The questions were answered at a household level, except for those regarding the specific latrine type and sharing latrines, which were responded to at a latrine level since some households used more than one latrine.

### Water and sanitation facility definitions

The WHO defines five levels of access to water and sanitation, respectively, using similar terminology. The first (safely managed) and second (basic) levels of the ladder are grouped as ‘at least a basic service.’ This category is one of the trace indicators used for monitoring progress towards Sustainable Development Goals’ target 1.4 on universal access to basic services. A household is considered to have access to at least a basic water source when it has at least one improved source, an access that is protected from outside contamination, within 30 minutes of walking distance. For sanitation, this refers to access to at least one improved facility, a service that ensures separation of human excreta from human contact, which is not shared with other households [2].

### Data analysis

We used proportions and dispersion metrics to describe the demographics and access to WASH. For all analyses we used *R*, version 4.4.3 (R Core Team, Vienna, Austria).

Without considering missing values, we performed calculations to determine the continuous variables – median and interquartile range (the first and third quantiles) or mean (x̄) and standard deviation after testing for normality using the Shapiro-Wilk test. We calculated the frequency and percentage distribution of each category for the categorical variables. In the variables with multiple responses, we took the number of responses as the denominator.

A wealth index was developed for each household in Mopeia as described previously [10]. In brief, we used the Demographic and Health Survey method [11], and then we conducted robustness tests to determine the individual weight of its different components. We ranked households in quintiles.

## RESULTS

### Demographic information

We surveyed a total of 7462 households. The household size was x̄ = 5.41 [7]. The survey registered a total of 40 331 individuals, of whom 20 622 (51.13%) were females. Mopeia’s population has a skewed age distribution, with 6786 (16.83%) aged <5 years, 12 829 (31.81%) aged 5–15 years, and 20 716 (51.36%) aged >15 years. Regarding the wealth index, a quarter of the households were ranked in the least poor quintile (n = 10 400, 25.79%) and 5541 (13.74%) in the poorest quintile (**Table 1**).

**Table 1:** Mopeia population demographic characteristics

Characteristics	n (%)
Sex	
*Female*	20 622 (51.13)
*Male*	19 709 (48.87)
Age	
*<5*	6786 (16.83)
*5–15*	12 829 (31.81)
*>15*	20 716 (51.36)
Wealth index rank	
*Least poor*	10 400 (25.79)
*Less poor*	9225 (22.87)
*Moderately poor*	8257 (20.47)
*Poorer*	6908 (17.13)
*Poorest*	5541(13.74)

### Household access to water

A total of 4200 (56.29%) of households were using at least a basic water service (**Table 2**). Most water sources in Mopeia were improved (n = 5274, 70.68%). The main water source for cooking and hygiene in Mopeia was a borehole protected with a hand pump (n = 4037, 54.65%). About three-quarters of households (n = 5552, 74.40%) used a water source located within 30 minutes walking distance.

**Table 2:** Description of the household’s water access

Water access	n (%)
At least a basic water service*	
No	2793 (37.43)
*Unimproved source*	1343 (18.00)
*>30 min*	1078 (14.45)
*Unimproved source and >30 min*	372 (4.99)
Yes	4200 (56.29)
NA	469 (6.29)
**Main water source for cooking and hygiene**	
Improved	5274 (70.68)
*Borehole with protected hand pump outside the yard*	4037 (54.10)
*Fountain*	700 (9.38)
*Protected well outside the yard*	359 (4.81)
*Protected well inside the yard*	59 (0.79)
*Borehole with a protected hand pump inside the household*	41 (0.55)
*Piped water in the yard*	33 (0.44)
*Piped water in the neighbour’s yard/house*	32 (0.43)
*Piped water in the house*	11 (0.15)
*Rainwater*	2 (0.03)
Unimproved	1715 (22.98)
*Unprotected well outside the household*	1033 (13.84)
*Water from the river*	434 (5.82)
*Lagoon/lake*	128 (1.72)
*Unprotected well inside the yard*	120 (1.61)
NA	473 (6.34)
Time to the water source in minutes	
*<10*	2126 (28.49)
*10–30*	3426 (45.91)
*30–60*	1193 (15.99)
*>60*	257 (3.44)
NA	460 (6.16)

NA – no answer

*At least one improved water source, which is less than 30 min away from the household.

### Household sanitation services

In Mopeia, only 116 (1.55%) of households fulfil at least a basic sanitation service criterion (**Table 3**). Over one half of the households (n = 4177, 55.98%) did not possess a latrine, and only a small fraction (n = 12, 0.16%) used improved and unimproved sanitation practices.

**Table 3:** Description of the household’s sanitation

Sanitation	n (%)
At least a basic sanitation service*	
No	6608 (88.56)
*Unimproved source*	6175 (82.75)
*Other than the latrine*	4177 (55.98)
*Latrine*	1998 (26.78)
*Unimproved and shared latrine*	412 (5.52)
*Shared latrine*	21 (0.28)
Yes	116 (1.55)
NA	738 (9.89)
**Sanitation service**	
Unimproved	6533 (87.55)
Improved	176 (2.36)
NA	738 (9.89)
Both	12 (0.16)
Prefer not to answer	3 (0.04)
**Latrine possession**	
No	4177 (55.98)
Yes	2547 (34.13)
NA	738 (9.89)
**Place of defecation of those who do not own a latrine (n = 4177)**	
Unimproved	4131 (98.90)
Improved	39 (0.93)
Both	4 (0.10)
Prefer not to answer	3 (0.07)
**Place of defecation specified (n = 4351)†**	
Improved	43 (0.99)
*The toilet is connected to the septic tank of the neighbouring house*	27 (0.67)
*Ventilated latrine with a slab from the neighbouring house*	12 (0.28)
*Unventilated latrine with a slab from the neighbouring house*	2 (0.05)
Unimproved	4305 (98.94)
*Bush with a hoe*	2689 (61.80)
*Open land defecation*	1148 (26.38)
*The traditional latrine of the neighbouring house*	379 (8.71)
*Open waste burning in the neighbouring house*	45 (1.03)
*Unimproved latrine of the neighbouring house*	43 (9.88)
*Other (specify): neighbour’s latrine*	1 (0.02)
Prefer not to answer	3 (0.07)
**Close to a water body**	
No	3525 (84.39)
Yes	606 (14.51)
Do not know	46 (1.10)
**Own latrine (n = 2547)**	
Unimproved	2402 (94.31)
Improved	137 (5.38)
Both	8 (0.31)
**Latrine type specified (n = 2598)‡**	
Unimproved	2440 (93.92)
*The traditional latrine of the neighbouring house*	2064 (79.45)
*Unimproved latrine*	361 (13.90)
*Open waste burning*	15 (0.58)
Improved	158 (6.10)
*The toilet is connected to a septic tank*	92 (3.54)
*Ventilated latrine with a slab*	37 (1.42)
*Unventilated latrine with a slab*	29 (1.12)
**Latrine shared with other households (n = 2598)‡**	
No	2148 (82.68)
Yes	446 (17.17)
Do not know	4 (0.15)
**Number of households sharing a latrine (n = 2598)**	
1	220 (8.47)
2–5	200 (7.70)
≥6	26 (1.00)
NA	2152 (82.83)

NA – no answer

*At least one improved sanitation service which is not shared with other households.

†Multiple-choice, the N is the total number of answers from 4177 households.

‡Latrine level; 44 households own more than one latrine and of those households seven own three toilets.

Among the households without latrines, <1% (n = 39, 0.93%) had an improved sanitation service. Regarding the specific place of defecation, almost two-thirds (n = 2689, 61.80%) defecated in the bush and covered it using a hoe and 1148 (26.38%) practised open land defecation. Most households without a latrine defecated at a distance greater than 10 m away from any water body (n = 3525, 84.39%).

Among households that possess a latrine, 145 (5.69%) had at least one improved latrine, while traditional latrine was the most common type (n = 2064, 79.45%); the latter is considered unimproved as it does not prevent contact with human excreta. Most latrines were not shared with other households (n = 2148, 82.68%).

### Cholera outbreak

In 2022, following the flooding caused by the Gombe cyclone [12], a cholera outbreak affected Mopeia and significantly disrupted the development of the BOHEMIA project. Further, 584 cases were registered from the middle of February to May in just three health units (Mopeia Sede, Chimuara, and Gulamo) in the district [13,14]. The highest number of cases was reported in Mopeia Sede and peaked in March. A temporary rehydration facility was set up. Neither a cholera rapid diagnostic test (RDT) or culture was performed.

**Figure 2:**
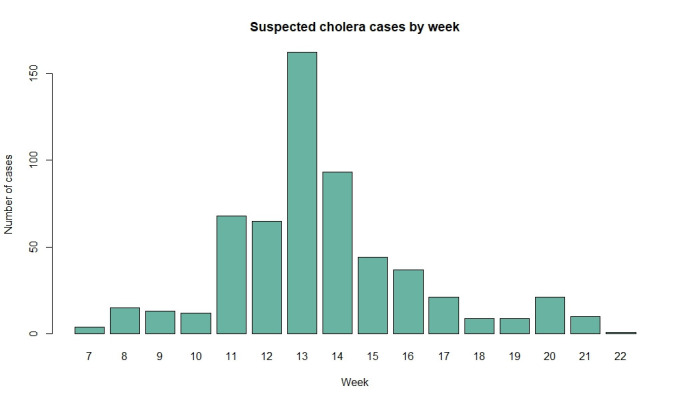
Suspected cholera cases by week.

The first cholera case was reported in the middle of February (**Figure 2**). The numbers increased significantly in March, when 321 (54.79%) of the cases were notified. The peak of the outbreak occurred at the end of March (13th epidemiological week), with 162 cases, and they gradually declined to 11 cases (1.88%) by the end of May. Most cases were female (n = 307, 52.57%) and aged ≥15 years (n = 330, 56.06%). The vast majority (n = 538, 92.92%) presented with watery ‘rice water’ diarrhoea and slightly more than half (n = 302, 52.43%) referred vomiting. Most cases had moderate (n = 227, 39.21%) or mild (n = 220, 38.00%) dehydration, whereas 132 (22.78%) were severely dehydrated. Abdominal cramps were the most frequent accompanying symptoms (n = 246, 43.67%). Oral rehydration salts were the most common treatment type (n = 427, 62.70%), followed by intravenous hydration (n = 157, 23.05%), and antibiotics (n = 97, 14.24%). Most patients were admitted for one day (n = 411, 70.38%). Malaria RDTs were performed on 203 (34.76%) of patients, and the RDT positivity rate was 54.19%. Despite only one cholera death reported in the inpatient department, verbal autopsy data from the study area conducted throughout the BOHEMIA trial show that at least 16 deaths in the community during the 2022 rainy season can be attributed to cholera.

## DISCUSSION

The availability, accessibility and sustainable management of water and sanitation is key to poverty elimination. Due to the large variation in access within Mozambique, a description of the most vulnerable areas is crucial for planning interventions [2].

The proportion of households with basic water service in Mopeia was similar to that estimated in both rural Mozambique and SSA. It is too slow to reach universal coverage of basic water services by 2030. Surprisingly, surface water (river, lagoon or lake), the least safe water source, was less frequent compared to SSA as a whole [6]. This is important given the high risk of contamination associated with these sources in underserved regions. Women and children, who are usually responsible for collecting water, are especially vulnerable while walking to open sources far away from their homes. Furthermore, a distant water source reduces the mothers’ available time for economic labour as well as personal and childcare, which can have important consequences in nutrition and safety. In the case of children, a distant water source may contribute to school absenteeism and social risks.

The coverage of basic sanitation was worryingly low in Mopeia, lower than WHO estimates for any country in 2022 [6]. Regardless of whether they had a latrine, most households used unimproved sanitation facilities. In Mopeia, the practice of open defecation doubled that reported in rural SSA and Mozambique. This category includes defecating in the bush and covering it with a hoe, land defecation and waste burning. Not only does open defecation have a relationship with several infectious diseases, but it also puts women and children at higher risk of social harm.

In Mopeia, having unimproved water access and sanitation facilities was the main factor for those services not reaching the minimum classification as ‘basic.’ Long distances to the water source and sharing latrines among households were less relevant, especially for sanitation. Understanding the underlying factors contributing to the classification of WASH services is crucial for planning the most effective public health intervention. Regarding water, behavioural interventions and community sensitisation campaigns could improve collection practices. However, factors such as infrastructure and road security may require addressing in places such as Mopeia, where an essential proportion of the population uses distant water sources.

The description of the 2022 cholera outbreak highlights the importance of appropriate access to safe WASH practices in Mopeia. In addition to the outbreak´s direct effects on morbidity, it severely disrupted the progression of key programmes and studies ongoing in Mopeia at that time. Current water and sanitation services in Mopeia require intervention to reduce the probability and impact of cholera and other WASH-related diseases.

Our descriptive study has some limitations. First, most of the data from WHO regarding WASH that we used as a reference to compare our percentages was obtained with approximations. Moreover, the representativeness of our sample could be compromised since this subpopulation is taken from a study that has exclusion criteria. Regarding the analysed variables, only WASH facilities that were used as a household were considered, but there are many that some individuals could use, for example, at school, that were not recorded. Finally, the answers given by the participants were not verified. However, despite such limitations, this study provides valuable indicators that could be leveraged by national programs in the assessment of access and usage of WASH practices in rural Mozambique.

## CONCLUSIONS

Data on water found in Mopeia was similar to that estimated for rural Mozambique and SSA but the percentages for basic sanitation services were worryingly lower, especially due to the use of unimproved sanitation facilities. Mopeia is still far from achieving universal safe water and sanitation coverage by 2030 and remains prone to outbreaks and has a high burden of WASH-related diseases.
